# Adverse reaction signals mining and hemorrhagic signals comparison of ticagrelor and clopidogrel: A pharmacovigilance study based on FAERS

**DOI:** 10.3389/fphar.2022.970066

**Published:** 2022-10-03

**Authors:** Shu Tang, Zhanshen Wu, Liqing Xu, Qiang Wen, Xiaojian Zhang

**Affiliations:** ^1^ Department of Pharmacy, The First Affiliated Hospital of Zhengzhou University, Zhengzhou, China; ^2^ Institute of Clinical Pharmacology, Zhengzhou University, Zhengzhou, China

**Keywords:** ticagrelor, clopidogrel, FAERS, pharmacovigilance, adverse events, hemorrhagic signals

## Abstract

**Background:** Ticagrelor and clopidogrel are commonly used antiplatelet agents, and we conducted a pharmacovigilance analysis using the Food and Drug Administration Adverse Event Reporting System (FAERS) to provide a reference for safe and reasonable clinical use.

**Methods:** Data were collected in FAERS from 2012 Q1 to 2022 Q2 for data cleaning. We used system organ classes (SOCs) and prefer terms (PTs) from the Medical Dictionary of Regulatory Activity (MedDRA version 25.1). Adverse event reports were retrieved at the PT level. Adverse reaction (ADR) signals of ticagrelor and clopidogrel were mined by calculating reporting odds ratios (ROR), proportional reporting ratios (PRR), information component (IC) and empirical Bayesian geometric mean (EBGM). After that, further analysis of the hemorrhagic signals and their clinical information were performed.

**Results:** The number of ADR reports where the primary suspect (PS) drugs were 15,133 for ticagrelor and 23,860 for clopidogrel. Significant ADR signals were identified by the SOC analysis for ticagrelor including cardiac disorders (ROR 4.87, PRR 4.46), respiratory disorders (ROR 2.45, PRR 2.28), and vascular disorders (ROR 2.22, PRR 2.16). Clopidogrel included blood disorders (ROR 2.86, PRR 2.77), vascular disorders (ROR 2.71, PRR 2.61), and cardiac disorders (ROR 2.29, PRR 2.22). At the PT level, the more frequent ADR signals for ticagrelor were dyspnoea, contusion, and haemorrhage, while clopidogrel were gastrointestinal haemorrhage, anaemia, and drug interaction. The hemorrhagic signals of both were mainly focused on the SOC level of gastrointestinal disorders, injury disorders and vascular disorders and nervous system disorders. The death and life-threatening rate of ticagrelor was 7.76 percentage higher than that of clopidogrel.

**Conclusion:** Clinicians need to pay attention to not only common ADRs but also be alert to new ADR signals when choosing to use ticagrelor and clopidogrel. This study provides a reference for the reasonable and safe clinical use of ticagrelor and clopidogrel.

## Introduction

Ticagrelor and clopidogrel are commonly used P2Y12 receptor inhibitors in clinical practice. In patients with acute coronary syndrome (ACS) and after percutaneous coronary intervention (PCI), dual antiplatelet therapy with aspirin combined with one of these is the basis of antithrombotic therapy. The mechanism differs between the two, with ticagrelor exerting its antiplatelet effect by reversibly and non-competitively directly inhibiting the P2Y12 receptor and limiting the ADP-mediated conversion of glycoprotein IIb/IIIa to the activated form (Capodanno et al., 2010). Clopidogrel, on the other hand, irreversibly blocks the P2Y12 receptor, thereby exerting its antiplatelet effect (Hollopeter et al., 2001).

Ticagrelor was approved for marketing by the U.S. Food and Drug Administration (FDA) on 20 July 2011, and clopidogrel was approved for marketing in June 1998, and ADRs were gradually reported during the clinical application of both drugs. Common adverse effects of ticagrelor include bradycardia and AV block, dyspnea, and risk of bleeding (Gurbel et al., 2009; Goldberg et al., 2015; Scirica et al., 2018; Pujade et al., 2020; Escaned et al., 2021). Clopidogrel resistance occurs in approximately 30% of patients (Tantry et al., 2018; Ma et al., 2021). Common adverse reactions of clopidogrel are risk of bleeding, gastrointestinal complications, rash, fever and neutropenia (Doogue et al., 2005; Kang et al., 2015; Chan et al., 2019). A study that was based on FAERS database conducted by Serebruany VL et al. at the annual meeting of the European Society of Cardiology (ESC 2017) demonstrated significantly higher ticagrelor-related mortality than clopidogrel and prasugrel, which was not consistent with the results of previous PLATO study (Cannon et al., 2010). At the same time, due to the lack of sufficient evidence-based data on the efficacy and safety of ticagrelor and clopidogrel, there is still some confusion among clinicians regarding the choice of ticagrelor or clopidogrel.

In this study, the latest reported data from FAERS database were used to perform pharmacovigilance analysis of ticagrelor and clopidogrel to provide a reference for safe and reasonable clinical use.

## Materials and methods

### Data sources and procedures

The data for this study was obtained from the FAERS database of ADR reports from the first quarter of 2012 through the second quarter of 2022. The FEARS database is a publicly available database of self-reported ADRs from healthcare professionals, drug manufacturers, and patients in many countries around the world, with data updated quarterly (Zhai et al., 2019).

We imported all data into SQL Server 2019 to build the ADR database. To ensure that there was no duplicate data, we performed a two-step deduplication process (Omar et al., 2021). The data was first normalized and cleaned, and all duplicate rows were removed. After that, if the CASEID and FDA_DT were the same, deduplication was performed based on the latest FDA_DT (Hu et al., 2020). The ADRs with ROLE_COD listed as PS were further screened as the background basis for the whole study. The search terms for ticagrelor were BRILINTA, TICAGRELOR, BRILIQUE and AZD6140, and for clopidogrel were CLOPIDOGREL and PLAVIX.

ADRs were classified and described according to the PT and the SOC in the International MedDRA, version 25.1 (Peng et al., 2020).

### Statistical analysis

ROR and PRR were used in the proportional imbalance method for data mining (Evans et al., 2001; van Puijenbroek et al., 2002). The larger the ROR and PRR were, the stronger the ADR signal was, indicating a stronger statistical relationship between the target drug and the target ADR. The ADR signals were significant if a ≥ 3, ROR or PRR ≥ 2.0 and 95% confidence interval (95% CI) value exceeds 1.0. To reduce false-positive ADR signals, we also used EBGM and IC to confirm the ADR signals we found (Bate et al., 1998; Szarfman et al., 2002; Karahoca, 2012). The equations and criteria for the four algorithms are shown in Table 1 (Shao et al., 2021; Zhou et al., 2021). We used R 4.2.1 software to perform the statistical analysis of the data.

**TABLE 1:** Summary of four algorithms used for signals detection.

Algorithms	Equation	Criteria
ROR	ROR = ad/bc	ROR ≥ 2
	95% CI = e^ln(ROR)±1.96(1/a+1/b+1/c+1/d)^0.5^	95% CI > 1
PRR	PRR = a (c + d)/(a + b)/c	PRR ≥ 2
	χ^2^ = [(ad−bc)^2](a + b + c + d)/[(a + b) (c + d) (a + c) (b + d)]	χ^2^ ≥ 4
BCPNN	IC = log_2_(a(a+b + c + d)/(a+b)/(a+c))	IC025 > 0
	IC025 = e^ln(IC)−1.96(1/a+1/b+1/c+1/d)^0.5^	
MGPS	EBGM = a(a + b + c + d)/[(a+b) (a+c)]	EBGM05 > 2
	EBGM05 = e^ln(EBGM) −1.64(1/a+1/b+1/c+1/d)^0.5^	

## Result

### ADR reports and clinical information

Finally, we obtained 10252782 reports of PS drugs, and 15,133 and 23,860 ADRs of ticagrelor and clopidogrel, respectively. The clinical information of the two drugs are shown in Table 2. The proportion of male patients was slightly higher for ticagrelor (59.59%) than for clopidogrel (46.90%), but clopidogrel had a high value of missing sex (19.21%). Ticagrelor was mainly used in ACS, myocardial infarction and stent placement in patients with a median age of 67 years. Clopidogrel was primarily indicated for antiplatelet therapy, stent placement, and prophylaxis in patients with a median age of 72 years. The majority of patients in both were elderly patients between the ages of 65–84.

**TABLE 2:** ADE reports and clinical information.

	Ticagrelor	Clopidogrel
Total	15,133	23,860
Gender, n (%)		
Male	9,018 (59.59)	11,191 (46.90)
Female	5,272 (34.84)	8,085 (33.89)
Missing	843 (5.57)	4584 (19.21)
Age (years)		
Median (IQR)	67 (59-75)	72 (63–80)
<18	15 (0.10)	66 (0.28)
18–64	4267 (28.20)	5,037(21.11)
65–84	5,352 (35.36)	9,982 (41.84)
≥85	455 (3.01)	2,241 (9.39)
Missing	5,044 (33.33)	6,534 (27.38)
Outcome		
Death	1,883 (16.57)	2,578 (11.67)
Life-Threatening	1,241 (10.92)	1,935 (8.76)
Hospitalization	5,771 (50.79)	11,535 (52.20)
Disability	315(2.77)	764(3.46)
Indication		
Acute coronary syndrome	3,305 (27.86)	811 (4.09)
Myocardial infarction	2.018 (17.01)	687 (3.46)
Stent placement	1,764 (14.87)	1,210 (6.10
Antiplatelet therapy	202 (1.70)	1637 (8.25)
Prophylaxis	62 (0.52)	935 (4.71)

**FIGURE 1:**
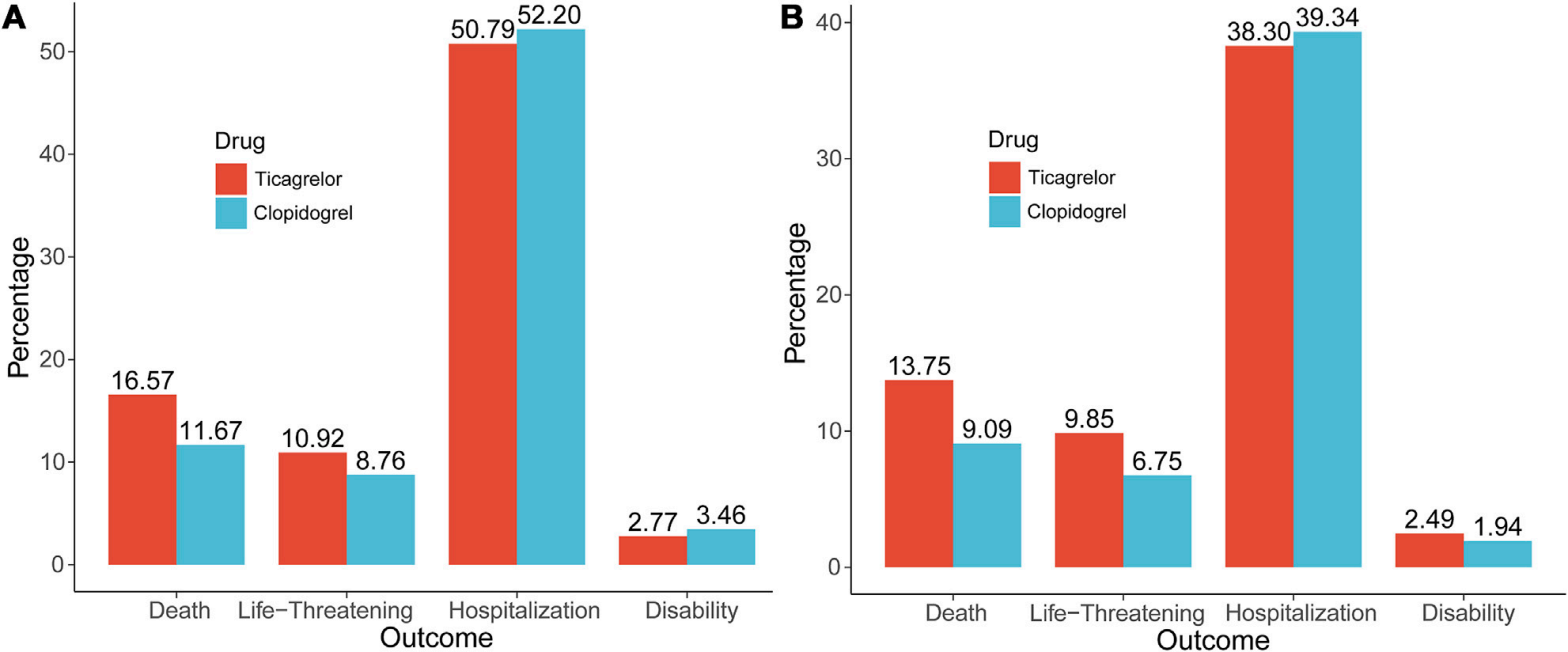
The Outcome indicators of ticagrelor and clopidogrel. **(A)** Overall outcome indicators; **(B)** Outcome indicators of hemorrhagic signals.

In addition, we also visualized the overall outcome metric data for ticagrelor and clopidogrel, as shown in Figure 1A. The overall lethality of ticagrelor (16.57%) was slightly higher than that of clopidogrel (11.67%), with a smaller difference in life-threatening, hospitalization and disability.

### System organ classes disproportionality analysis

In the disproportionate analysis of SOCs, the significant signals for ticagrelor were cardiac disorders (ROR 4.87, PRR 4.46), respiratory disorders (ROR 2.45, PRR 2.28), and vascular disorders (ROR 2.22, PRR 2.16). Significant signals for clopidogrel were blood and lymphatic system disorders (ROR 2.86, PRR 2.77), vascular disorders (ROR 2.71, PRR 2.61), and cardiac disorders (ROR 2.29, PRR 2.22). As shown in Table 3, cardiac disorders and vascular disorders were common to both.

**TABLE 3:** Significant safety signals on the SOC level.

SOC	ROR (95%CI)	PRR (χ^2^)	IC (IC025)	EBGM (EBGM05)
Ticagrelor				
Cardiac disorders	4.87 (4.72–5.02)	4.46 (12479.14)	2.15 (2.08)	4.44 (4.33)
Respiratory disorders	2.45 (2.38–2.52)	2.28 (3619.78)	1.19 (1.15)	2.28 (2.22)
Vascular disorders	2.22 (2.12–2.32)	2.16 (1247.42)	1.11 (1.06)	2.16 (2.08)
Clopidogrel				
Blood and lymphatic system disorders	2.86 (2.77–2.96)	2.77 (4120.12)	1.46 (1.41)	2.76 (2.68)
Vascular disorders	2.71 (2.63–2.79)	2.61 (4367.90)	1.38 (1.34)	2.60 (2.53)
Cardiac disorders	2.29(2.22–2.36)	2.22 (2880.23)	1.15 (1.11)	2.22 (2.16)

### Adverse reaction frequency analysis

We performed a deeper analysis, the disproportionality analysis at the PT level. PTs related to ticagrelor and clopidogrel indications were removed from the analysis and ranked in descending order of the frequency and ROR of PTs. In Table 4, the top significant safety signals for ticagrelor and clopidogrel are shown separately, while we compared them with the adverse reactions spelled out in the drug instructions, using * to mark those not mentioned in the instructions. The 95% CI for ROR only shows the lower limit of the 95% two-sided CI of the ROR.

**TABLE 4:** Top significant signals on the PT level (*: The instruction does not mention).

PT	SOC	Freq	ROR (95%CI)	PRR (χ^2^)
Ticagrelor (Sorted by frequency)				
Dyspnoea	Respiratory, thoracic and mediastinal disorders	2,359	5.96 (5.72)	5.69 (9132.68)
Contusion	Injury, poisoning and procedural complications	717	10.34 (9.60)	10.18 (5873.07)
Haemorrhage	Vascular disorders	488	7.20 (6.58)	7.12 (2550.54)
Intentional product misuse*	Injury, poisoning and procedural complications	421	5.10 (4.63)	5.06 (1356.83)
Anaemia	Blood and lymphatic system disorders	417	7.44 (6.75)	7.38 (2279.22)
Product use issue	Injury, poisoning and procedural complications	379	2.60 (2.35)	2.58 (367.69)
Gastrointestinal haemorrhage	Gastrointestinal disorders	369	5.43 (4.90)	5.391312.01)
Blood pressure increased	Investigations	256	2.33 (2.06)	2.32 (192.68)
Ticagrelor (Sorted by frequency) Dyspnoea	Respiratory, thoracic and mediastinal disorders	2,359	5.96 (5.72)	5.69 (9132.68)
Cerebral haemorrhage	Nervous system disorders	240	9.20 (8.10)	9.15 (1723.77)
Haemoglobin decreased	Investigations	228	3.07 (2.69)	3.06 (316.28)
Clopidogrel				
Gastrointestinal haemorrhage	Gastrointestinal disorders	3162	27.58 (26.59)	26.50 (73189.45)
Anaemia	Blood and lymphatic system disorders	1622	6.29 (5.99)	6.18 (6959.36)
Drug interaction	General disorders and administration site conditions	1239	6.39 (6.04)	6.30 (5459.49)
Cerebral haemorrhage	Nervous system disorders	1134	24.94 (23.48)	24.60 (24293.19)
Haemorrhage	Vascular disorders	1028	8.35 (7.85)	8.25 (6436.63)
Haematochezia	Gastrointestinal disorders	815	11.84 (11.04)	11.72 (7789.85)
Melaena	Gastrointestinal disorders	788	27.71 (25.78)	27.44 (18877.33)
Multiple injuries*	Injury, poisoning and procedural complications	681	242.68 (220.89)	240.57 (104118.10)
Rectal haemorrhage	Gastrointestinal disorders	678	11.82 (10.95)	11.72 (6479.08)
Epistaxis	Respiratory, thoracic and mediastinal disorders	666	6.45 (5.97)	6.41 (2997.94)
Ticagrelor (Sorted by ROR)				
Paroxysmal atrioventricular block*	Cardiac disorders	3	139.19 (40.7)	139.18 (349.84)
Cheyne-Stokes respiration*	Respiratory, thoracic and mediastinal disorders	12	86.06 (47.40)	86.04 (909.42)
Haemorrhage coronary artery	Cardiac disorders	3	65.73 (20.20)	65.73 (176.51)
Sinus arrest*	Cardiac disorders	59	64.72 (49.60)	64.63 (3416.23)
Gastrointestinal vascular malformation*	Gastrointestinal disorders	3	63.95 (19.70)	63.95 (171.95)
Ventricle rupture*	Cardiac disorders	4	55.35 (20.00)	55.35 (199.47)
Rhythm idioventricular*	Cardiac disorders	10	50.57 (26.60)	50.56 (456.52)
Dressler’s syndrome*	Cardiac disorders	4	50.08 (18.20)	50.08 (180.90)
Sinoatrial block*	Cardiac disorders	16	44.93 (27.10)	44.91 (649.89)
Clopidogrel				
Preternatural anus*	Congenital, familial and genetic disorders	9	965.82 (297.42)	965.71 (2668.74)
Capillary fragility test*	Investigations	7	429.24 (150.56)	429.20 (1495.22)
Metallosis of globe*	Injury, poisoning and procedural complications	3	429.22 (86.63)	429.20 (640.81)
Cullen’s sign*	Skin and subcutaneous tissue disorders	6	257.54 (93.60)	257.52 (958.22)
Orbital compartment syndrome*	Eye disorders	7	250.39 (98.58)	250.37 (1098.07)
Insulin autoimmune syndrome*	Immune system disorders	54	246.73 (176.54)	246.56 (8388.07)
Multiple injuries*	Injury, poisoning and procedural complications	681	242.68 (220.89)	240.57 (104118.10)
CYP2C19 polymorphism*	Congenital, familial and genetic disorders	5	238.46 (79.91)	238.45 (760.02)
Oesophageal intramural haematoma*	Gastrointestinal disorders	15	222.04 (119.04)	222.00 (2175.07)
Haemorrhagic thyroid cyst*	Endocrine disorders	3	214.61 (53.67)	214.60 (425.21)

The frequent adverse safety signals for ticagrelor were dyspnoea, contusion, and haemorrhage, the largest ROR values were paroxysmal atrioventricular block, tooth pulp haemorrhage and cheyne-Stokes respiration. The adverse signals not mentioned in the instructions were intentional product misuse, paroxysmal atrioventricular block, tooth pulp haemorrhage, cheyne-Stokes respiration, sinus arrest, gastrointestinal vascular malformation, ventricle rupture, rhythm idioventricular, dressler’s syndrome, sinoatrial block. The frequent adverse reaction signals of clopidogrel were gastrointestinal haemorrhage, anaemia and drug interaction. The signals of adverse reactions not mentioned in the instructions were preternatural anus, capillary fragility test, metallosis of globe, cullen’s sign, orbital compartment syndrome, insulin autoimmune syndrome, multiple injuries, CYP2C19 polymorphism, oesophageal intramural haematoma and haemorrhagic thyroid cyst. The analysis of real-world study based on the FAERS database also provides great reference value for the revision of the instructions for ticagrelor and clopidogrel.

### Comparison of hemorrhagic signals

The main effect of ticagrelor and clopidogrel were antiplatelet, and our deeper comparison assessed their significant adverse hemorrhagic signals. Ticagrelor had a total of 125 PT level hemorrhagic ADR signals, and clopidogrel had a total of 256, mainly focused on gastrointestinal disorders, injury disorders, nervous system disorders and vascular disorders. The overall incidence of bleeding events was slightly lower with ticagrelor than with clopidogrel (9.70% vs. 12.65%). Both gastrointestinal disorders and nervous system disorders dominated. As shown in Table 5, for a single SOC item we list the three PTs with the highest frequency. The most frequent of ticagrelor’s gastrointestinal disorders were gastrointestinal haemorrhage, rectal haemorrhage and gastric ulcer, and those for clopidogrel were gastrointestinal haemorrhage, haematochezia and melaena. Table 5 allows us to directly compare the strength of the hemorrhagic adverse reaction signals, and also greatly facilitates the comparison and deeper excavation of the major hemorrhagic adverse reaction signals of both.

**TABLE 5:** Major hemorrhagic signals.

SOC (n, %)	PT (Top 3)	ROR (95%CI)	PRR (χ^2^)	IC (IC025)	EBGM (EBGM05)
Ticagrelor					
Gastrointestinal disorders (42, 33.60)	Gastrointestinal haemorrha	5.43 (4.90)	5.39 (1312.01)	2.42 (2.18)	5.36 (4.92)
	Rectal haemorrhage	3.96 (3.33)	3.95 (278.39)	1.98 (1.66)	3.93 (3.40)
	Gastric ulcer	8.34 (6.93)	8.34 (6.93)	8.34 (6.93)	8.34 (6.93)
Injury, poisoning and procedural complications (17, 13.60)	Contusion	10.34 (9.60)	10.18 (5873.07)	3.33 (3.09)	10.07 (9.46)
	Subdural haematoma	7.01 (5.63)	7.00 (413.23)	2.80 (2.25)	6.95 (5.79)
	Post procedural haemorrhage	5.96 (4.50)	5.95 (200.28)	2.56 (1.93)	5.91 (4.67)
Nervous system disorders (15, 12.00)	Cerebral haemorrhage	9.20 (8.10)	9.15 (1723.77)	3.18 (2.80)	9.06 (8.14)
	Haemorrhage intracranial	14.69 (12.50)	14.63 (1983.49)	3.85 (3.29)	14.39 (12.62)
	Haemorrhagic stroke	11.00 (8.69)	10.98 (626.67)	3.44 (2.72)	10.85 (8.91)
Vascular disorders (8, 6.40)	Haemorrhage	7.20 (6.58)	7.12 (2550.54)	2.82 (2.58)	7.07 (6.56)
	Haematoma	4.91 (4.02)	4.90 (299.73)	2.29 (1.88)	4.88 (4.13)
	Shock haemorrhagic	7.90 (5.94)	7.89 (285.92)	2.97 (2.23)	7.82 (6.16)
Clopidogrel					
Gastrointestinal disorders (69, 26.95)	Gastrointestinal haemorrhage	27.58 (26.59)	26.50 (73189.45)	26.50 (73189.45)	26.50 (73189.45)
	Haematochezia	11.84 (11.04)	11.84 (11.04)	11.84 (11.04)	11.84 (11.04)
	Melaena	27.71 (25.58)	27.44 (18877.33)	4.69 (4.36)	25.85 (24.33)
Injury, poisoning and procedural complications (35, 13.67)	Multiple injuries	242.68 (220.89)	240.57 (104118.13)	7.27 (6.62)	154.52 (142.82)
	Contusion	4.42 (4.07)	4.42 (4.07)	4.42 (4.07)	4.42 (4.07)
	Subdural haematoma	19.54 (17.67)	19.45 (6681.97)	4.22 (3.82)	18.65 (17.14)
Nervous system disorders (26, 10.16)	Cerebral haemorrhage	24.94 (23.48)	24.60 (24293.19)	4.54 (4.27)	23.32 (22.17)
	Haemorrhage intracranial	12.48 (10.99)	12.45 (2517.64)	3.60 (3.17)	12.13 (10.91)
	Hemiparesis	8.26 (7.15)	8.24 (1187.11)	3.02 (2.62)	8.11 (7.19)
Vascular disorders (19, 7.42)	Haemorrhage	8.35 (7.85)	8.25 (6436.63)	3.02 (2.84)	8.11 (7.70)
	Haematoma	13.38 (12.21)	13.31 (5212.45)	3.69 (3.37)	12.94 (11.98)
	Shock haemorrhagic	12.98 (10.98)	12.95 (1520.71)	3.66 (3.10)	12.60 (10.96)

After that, this study went deeper to compare the clinical information of the hemorrhagic signals, as shown in Table 6. In total, there were 3,640 patients with ticagrelor and 13,099 patients with clopidogrel. Regarding the gender of the patients, the number of males was much higher than that of females in both, but clopidogrel had a higher missing gender values, 22.03% vs. 4.12%. In terms of age, the median value of ticagrelor (68 years) was smaller than that of clopidogrel (73 years), and both drugs were used to treat the largest proportion of patients between 65 and 84 years. Ages from both also had large missing values, 27.67% for ticagrelor and 25.25% for clopidogrel.

**TABLE 6:** Clinical information associated with hemorrhagic signals.

	Ticagrelor	Clopidogrel
Total	3640	13,099
Gender, n (%)		
Male	2153 (59.15)	6162 (47.04)
Female	1337 (36.73)	4052 (30.93)
Missing	150 (4.12)	2885 (22.03)
Age (years)		
Median (IQR)	68 (60–76)	73 (63–81)
<18	3 (0.08)	29 (0.22)
18–64	1001 (27.50)	2662 (20.32)
65–84	1471 (40.41)	5818 (44.42)
≥85	158 (4.34)	1445 (11.03)
Missing	1007 (27.67)	3145 (24.01)
Outcome		
Death	614 (13.75)	1,688 (9.09)
Life-Threatening	440 (9.85)	1,254 (6.75)
Hospitalization	1,710 (38.30)	7,303 (39.34)
Disability	111 (2.49)	361 (1.94)

We then counted the outcome indicators for all patients, as shown in Figure 1B, and the lethality rate was higher for ticagrelor (13.75%) than for clopidogrel (9.09%), with a difference of 4.66% points. The life-threatening rate was also higher for ticagrelor (9.85%) than for clopidogrel (6.75%), with a difference of 3.10% points. The difference between the two hospitalization rates (38.30% vs. 39.34%), was not much. Death and life-threatening events were the more serious adverse outcome events, and ticagrelor was 7.76% points higher than clopidogrel.

## Disscussion

Based on data from the FAERS database from 2012Q1 to 2022Q2 quarters, the study used ROR and PRR as the primary assays, IC and EBGM as confirmation methods to perform a pharmacovigilance analysis of ticagrelor and clopidogrel to provide a reference for safe and reasonable clinical use of the drugs. ADR signals and hemorrhagic events provided the real-world based reference value.

For ticagrelor and clopidogrel, it is also important to understand the clinical application scenarios for which they are better suited. In patients with acute myocardial infarction, ticagrelor was significantly more effective than clopidogrel (*p* <0.05), and the incidence of ADR was significantly lower than that of clopidogrel (*p* <0.05). The effect of ticagrelor on acute myocardial infarction patients is significantly better than clopidogrel, and has higher safety (Ma et al., 2020). Ticagrelor has beneficial effects in clinical application, while it has a higher incidence of dyspnoea and major bleeding compared to clopidogrel (Steiner et al., 2013).

In this study, we concluded that the overall mortality of ticagrelor was higher than that of clopidogrel (16.57% vs. 11.67%), which is not consistent with previous research. For patients with ACS, the proportion of death and life-threatening events with ticagrelor was more than with clopidogrel (25.54% vs. 22.28%). For patients with stent placement, the proportion of death and life-threatening events with ticagrelor was less than with clopidogrel (11.61% vs. 14.21%). For patients with myocardial infarction, the proportion of death and life-threatening events with ticagrelor was lower than with clopidogrel (19.62% vs. 21.91%). The choice of ticagrelor or clopidogrel in different clinical scenarios can reduce the incidence of death and life-threatening events to a certain extent.

The FAERS database also has certain limitations, such as duplicate reporting, incomplete reporting, irregular reporting, and mixed reporting of indications and adverse reactions. We cleaned the collected data more thoroughly, so that the quality of the data obtained was more reliable and the analysis results were more accurate.

### System organ classes level analysis

In the disproportionate analysis of SOC levels, ticagrelor focused on cardiac disorders, respiratory disorders, and vascular disorders, which was in high agreement with the PLATO study in which the most common adverse effects in patients were dyspnea and haemorrhage (Cannon et al., 2010). The adverse effect of bradycardia in cardiac disorders has also been a cause of great alarm (Turgeon et al., 2015; Pujade et al., 2020). Clopidogrel focused mainly on blood and lymphatic system disorders, vascular disorders, and cardiac disorders, which was also in high agreement with the most common haemorrhage and hematologic abnormalities in the instructions (Kohriyama et al., 2014). In the SOC level analysis, cardiac disorders were somewhat biased because the applicable disorders were also grouped into PTs.

### New adverse reaction signals

After obtaining the results of all PT level ADR signals for ticagrelor and clopidogrel, the signals were ranked according to their frequency and ROR, mainly focusing on gastrointestinal disorders. The higher the frequency was the more valuable is the excavation. After comparing with the drug instructions, it was found that both showed new ADR signals that were not mentioned in the instructions.

ADR signals not mentioned in the ticagrelor specification were intentional product misuse, paroxysmal atrioventricular block, tooth pulp haemorrhage, and Cheyne-Stokes respiration. The unmentioned intentional product misuse (ROR 5.10, PRR 5.06) and the mentioned product use issue (ROR 2.60, PRR 2.58) both suggested that the use of ticagrelor can be more problematic in patients, and if taken in strict accordance with medical advice, it may be possible to somewhat reduce the associated ADRs. ADR signals not mentioned in the clopidogrel instructions were multiple injuries, preternatural anus, capillary fragility test, metallosis of globe. Multiple injuries (ROR 242.68, PRR 240.57) had high frequency and strong signal and alert us to pay close attention to this adverse reaction while using clopidogrel.

### Comparison of hemorrhagic signals and clinical information

A deeper analysis was a summary of all significant hemorrhagic signals for both. It can be seen that bleeding events of ticagrelor occurred mainly in the gastrointestinal tract (33.60%) and injury, procedural complications (13.60%) and clopidogrel mainly in the gastrointestinal tract (26.95%) and injury, procedural complications (13.67%). Two clinical information analyses were performed in this study. The outcome events from the first clinical information are shown in Figure 1A, where ticagrelor was more lethal and more life-threatening than clopidogrel.

The second clinical information focused on all patients who experienced hemorrhagic adverse events because both drugs are antiplatelet agents and haemorrhage is their most common and predominant adverse effect. As shown in Figure 1B, the lethality and life-threatening rate of ticagrelor was 7.76% points higher than that of clopidogrel. The difference in hospitalization rates between the two was not much. By the above analysis, considering all significant hemorrhagic signals alone, ticagrelor produced higher rates of lethality and life-threatening events.

## Conclusion

In this study, the FAERS database was used to perform the pharmacovigilance analysis of ticagrelor and clopidogrel, and the ADR signals at the SOC and PT levels were detected using the disproportionality method, provided some complementary ADR signals that are not mentioned in the instructions. Then by further analysis of hemorrhagic events, ticagrelor produced higher rates of lethality and life-threatening events. Clinicians need to be aware of not only common ADRs but also new ADR signals when choosing to use ticagrelor and clopidogrel. This study provides a reference for the reasonable and safe clinical use of ticagrelor and clopidogrel.

## Data Availability

The Publicly available datasets can be found here: https://fis.fda.gov/extensions/FPD-QDE-FAERS/FPD-QDE-FAERS.html.
